# The effects of contemporary treatment of DCIS on the risk of developing an ipsilateral invasive Breast cancer (iIBC) in the Dutch population

**DOI:** 10.1007/s10549-023-07168-8

**Published:** 2023-11-14

**Authors:** Sena Alaeikhanehshir, Renée S.J.M. Schmitz, Alexandra W. van den Belt-Dusebout, Frederieke H. van Duijnhoven, Ellen Verschuur, Maartje van Seijen, Michael Schaapveld, Esther H. Lips, Jelle Wesseling, and Grand Challenge PRECISION Consortium

**Affiliations:** 1 Division of Molecular Pathology, the Netherlands Cancer Institute – Antoni van Leeuwenhoek Hospital, Plesmanlaan 121, Amsterdam, 1066 CX Netherlands; 2 Department of Surgical Oncology, the Netherlands Cancer Institute – Antoni van Leeuwenhoek Hospital, Amsterdam, Netherlands; 3 Borstkanker Vereniging Nederland, Utrecht, The Netherlands; 4 Department of Epidemiology, The Netherlands Cancer Institute, Amsterdam, the Netherlands; 5 Department of Pathology, the Netherlands Cancer Institute – Antoni van Leeuwenhoek Hospital, Amsterdam, the Netherlands; 6 Department of Pathology, Leiden University Medical Center, Leiden, the Netherlands

**Keywords:** Ductal carcinoma in situ, Invasive breast cancer, Surgery, Radiotherapy, Population-based cohort study, Breast cancer-screening

## Abstract

**Purpose:**

To assess the effects of contemporary treatment of ductal carcinoma in situ (DCIS) on the risk of developing an ipsilateral invasive breast cancer (iIBC) in the Dutch female population.

**Methods:**

Clinical data was obtained from the Netherlands Cancer Registry (NCR), a nationwide registry of all primary malignancies in the Netherlands integrated with the data from PALGA, the Dutch nationwide network and registry of histo- and cytopathology in the Netherlands, on all women in the Netherlands treated for primary DCIS from 2005 to 2015, resulting in a population-based cohort of 14.419 women. Cumulative iIBC incidence was assessed and associations of DCIS treatment type with subsequent iIBC risk were evaluated by multivariable Cox regression analyses.

**Results:**

Ten years after DCIS diagnosis, the cumulative incidence of iIBC was 3.1% (95% CI: 2.6–3.5%) in patients treated by breast conserving surgery (BCS) plus radiotherapy (RT), 7.1% (95% CI: 5.5–9.1) in patients treated by BCS alone, and 1.6% (95% CI: 1.3–2.1) in patients treated by mastectomy. BCS was associated with a significantly higher risk for iIBC compared to BCS + RT during the first 5 years after treatment (HR 2.80, 95% CI: 1.91–4.10%). After 5 years of follow-up, the iIBC risk declined in the BCS alone group but remained higher than the iIBC risk in the BCS + RT group (HR 1.73, 95% CI: 1.15–2.61).

**Conclusions:**

Although absolute risks of iIBC were low in patients treated for DCIS with either BCS or BCS + RT, risks remained higher in the BCS alone group compared to patients treated with BCS + RT for at least 10 years after DCIS diagnosis.

## Introduction

Ductal Carcinoma In Situ (DCIS) is considered a potentially pre-invasive lesion in the ductal-lobular system of the breast [1], which may progress into Invasive Breast Cancer (IBC) if left untreated. It remains uncertain in which patients DCIS remains indolent and in which DCIS will develop into invasive disease [2, 3]. Before the introduction of population breast cancer screening, DCIS was rarely diagnosed. Nowadays, DCIS accounts for roughly 20% of all newly diagnosed breast tumors [4–6]. The standard management for DCIS includes breast conserving surgery (BCS) often followed by radiotherapy (RT) and in in some countries like the US endocrine treatment is also administered. If BCS is not achievable i.e. due to the ratio of the lesion size to breast size, a mastectomy (MST), with or without direct reconstruction, can be performed [7]. Nonetheless, the treatment of patients with DCIS is widely debated since not all DCIS patients will experience survival benefit from invasive treatment [8–12].

Several previous studies have assessed the risk of developing a subsequent iIBC following locoregional therapy of DCIS [13–15]. We have reported results from a population-based nationwide cohort study with a median follow-up of 15.7 years that included 10.045 patients diagnosed from 1989 to 2004. In this cohort, 13.9% of patients treated with BCS alone developed a subsequent iIBC compared to 5.2% of patients treated by BCS + RT, and 1.1% of patients treated by MST developed a subsequent iIBC [15]. However, the population included in this study consisted of patients diagnosed with DCIS during 1989–2004 when adjuvant RT was not yet standard of care and the Dutch nationwide breast cancer screening program was not yet fully implemented [14, 15]. More recently, much lower rates of invasive recurrences have been reported in patients with DCIS treated with BCS [16, 17]. Therefore, the current study investigates the effect of more contemporary treatment for DCIS on invasive recurrence rates in a Dutch population-based nationwide cohort comprising patients diagnosed with DCIS from 2005 to 2015.

## Methods

### Patient selection

**Fig. 1:**
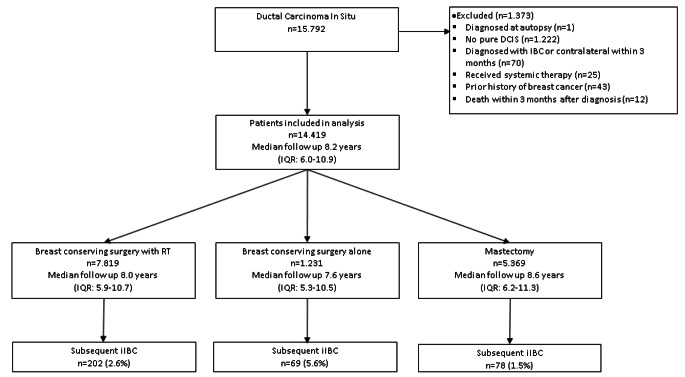
Flow diagram for patient selection and median follow-up by initial treatment type, RT, radiotherapy; IQR, interquartile range; iIBC, ipsilateral invasive breast cancer

This study includes all women treated for primary DCIS in the Netherlands from 2005 to 2015. Clinical data have been obtained from the Netherlands Cancer Registry (NCR), a nationwide registry of all primary malignancies in the Netherlands [18]. Data were subsequently linked with data from the nationwide network and registry of histology and cytopathology in the Netherlands (PALGA) [19]. PALGA data was used to check the medical history of breast cancer (including DCIS) and the presence of pure DCIS. Eligibility criteria were a diagnosis of pure DCIS, and surgical treatment with or without RT. Patients were excluded if DCIS diagnosis was determined at autopsy, if diagnosis of a subsequent iIBC occurred within three months of initial DCIS diagnosis, or if systemic therapy for initial DCIS diagnosis was administered. Systemic treatment is not administered as part of DCIS treatment in the Netherlands. If NCR reported an oncological medical history other than non-melanoma skin cancer, patients were also excluded. Ultimately the study cohort consisted of 14.419 patients with pure primary DCIS (see Fig. 1). The study was approved by the institutional review boards of NCR and PALGA.

### DCIS treatment and other characteristics

Data on age, year, histological grade, and treatment at DCIS diagnosis were provided by the NCR. Treatment for the primary DCIS lesion was categorized as (1) BCS + RT; (2) BCS alone; and (3) MST. All treatments for the ipsilateral breast within 3 months after DCIS diagnosis were considered primary treatment. If type of treatment for primary DCIS (n = 11) was unknown, treatment type information was extracted from pathology reports obtained from pathology laboratories through PALGA.

### Follow-up data

The occurrence of any iIBC at least 3 months after the primary DCIS diagnosis was ascertained based on NCR data. For patients initially treated with BCS, pathology data from PALGA were reviewed to identify ipsilateral MSTs (Interim MST) without a diagnosis of iIBC. Patients who underwent interim MST were classified as being initially treated by MST (n = 194). Data concerning subsequent iIBC and vital status was complete until February 1, 2020.

### Statistical analyses

Time at risk started 3 months after the diagnosis of primary DCIS and stopped at date of diagnosis of the event of interest (iIBC), date of death, or most last date of follow-up (February 1, 2020), whichever came first. Cumulative incidences were calculated for iIBC, with death considered as a competing risk. Contralateral IBCs and ipsilateral or contralateral DCIS recurrences were not considered events of interest and treatment for a contralateral event was not taken into account. The cumulative risk of a subsequent iIBC in patients with low-grade (grade I/II) versus patients with high-grade (grade III) DCIS was also determined, and for patients younger than 50 versus 50 years and older, in order to compare our results to prior publications [14]. P-values were based on competing risk regression [20], with time since DCIS diagnosis as time-scale and adjusted for age (continuous). Cox proportional hazards analyses, using age as primary time-scale and time since DCIS diagnosis as secondary time-scale (0–5, 5–10, and ≥ 10 years), were used to quantify the effects of different treatments on iIBC risk. We assessed interaction of treatment with age and grade. Proportional hazard assumptions were verified using graphical and residual-based methods. Potential confounders were included as confounders if the hazard ratio for treatment was changed by 10% or more in a model including the potential confounder(s) and treatment compared with a model with treatment alone. All statistical analyses were performed using STATA/SE 13.1 (StataCorp LP, College Station, TX). A two-sided P value less than 0.05 was considered statistically significant.

## Results

### Patient characteristics & treatment characteristics

The median age at DCIS diagnosis was 58 years (interquartile range (IQR) 51–66 years) and 83.8% was ≥ 50 years. Treatment consisted of BCS + RT in 54.2%, BCS alone in 8.5%, and of MST in 37.2%. Median follow-up was 8.2 years (IQR 6.0-10.9); 1.160 deaths (8.0%) patients died during follow-up. Table 1 shows patient characteristics, follow-up duration and number of iIBC cases by treatment type.

### Risk of ipsilateral invasive breast cancer

**Fig. 2:**
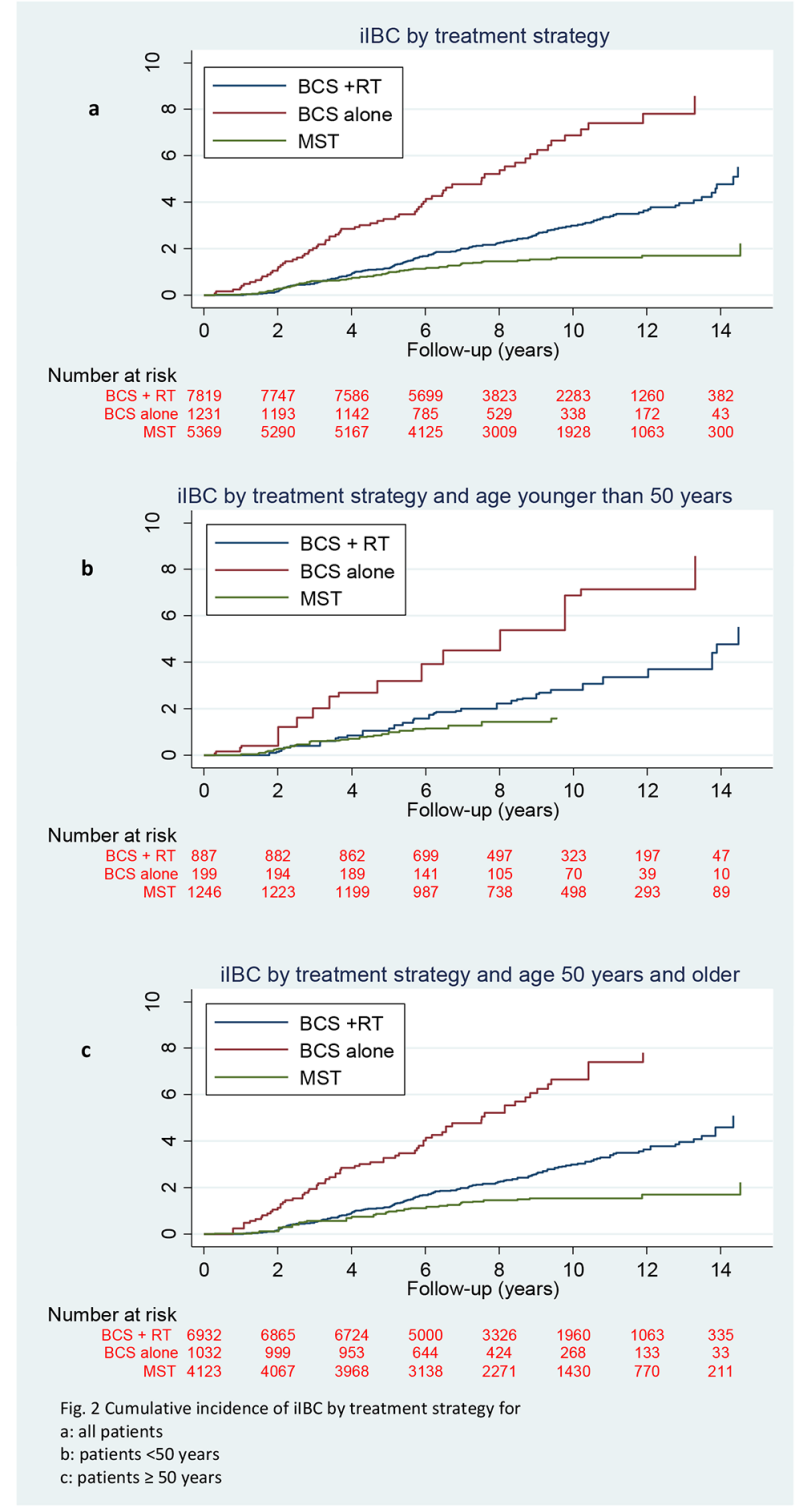
Cumulative incidence of iIBC by treatment strategy for a: all patients, b: patients, <50 years, c: patients ≥ 50 years

**Fig. 3:**
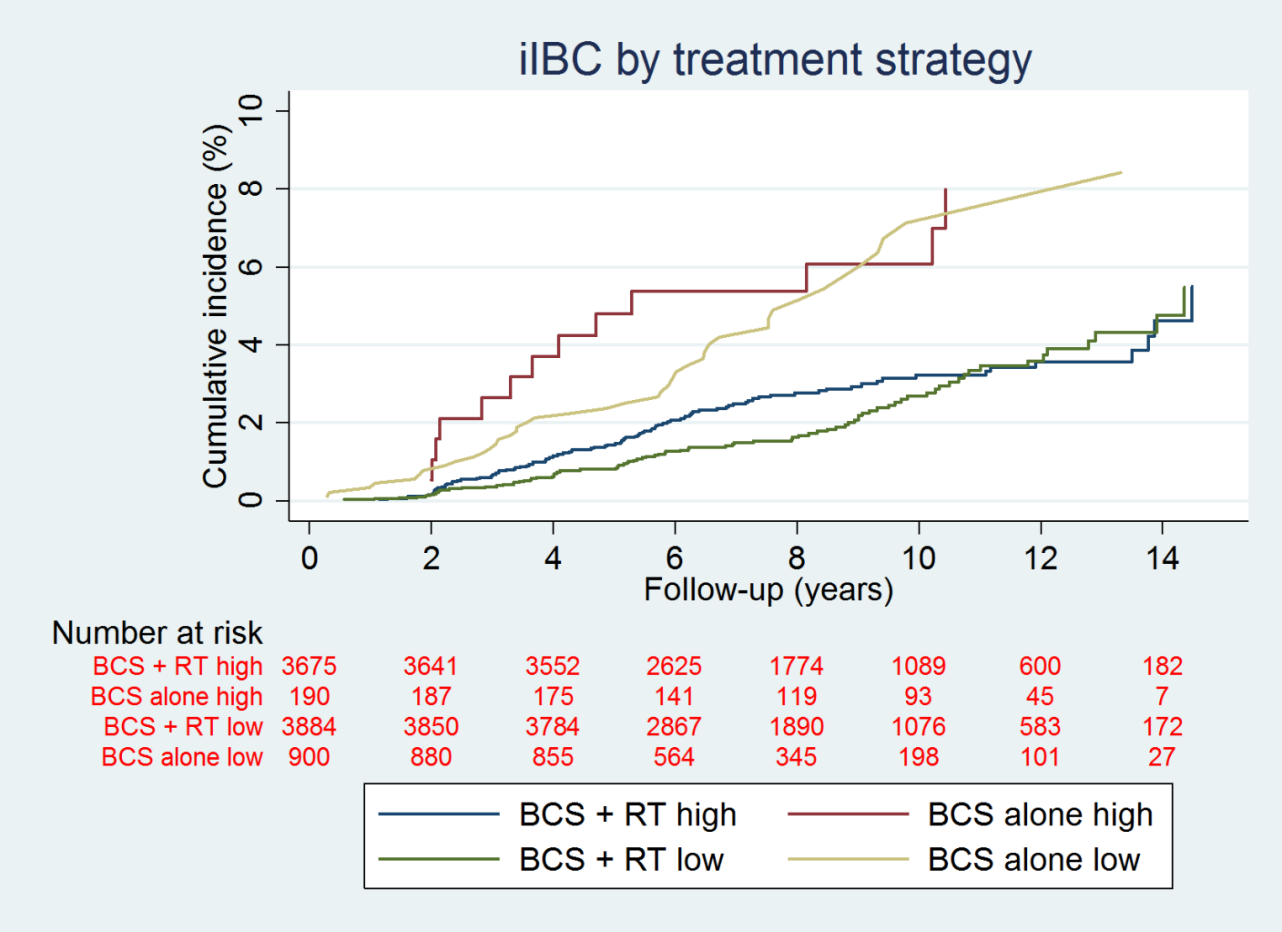
cumulative incidences of iIBC in patients with high and low grade DCIS by treatment strategy

Overall, 349 (2.4%) patients developed an iIBC with a median time to iIBC of 4.8 years (interquartile range 2.8-7.0 years). The 10-year cumulative incidence was 3.1% (95% CI: 2.6–3.5) in patients treated with BCS + RT, 7.1% (95% CI: 5.5–9.1) in patients treated by BCS alone, and 1.6% (95% CI: 1.3-2.0) in patients treated with MST (Fig. 2). The cumulative incidence of a subsequent iIBC was 3.2% (95% CI: 2.5–3.8) at 10 years in patients with grade III DCIS treated with BCS + RT and 2.7% (95% CI: 2.1–3.4) in patients with grade I/II DCIS treated with BCS + RT. In grade III DCIS patients treated with BCS alone the cumulative incidence of iIBC was 6.1% (95% CI: 3.2–10.2), versus a cumulative incidence of 7.1% (95% CI: 5.2–9.7) in grade I/II DCIS patients (Fig. 3). At 5 years, the cumulative incidence of subsequent iIBC was 2.1% (95% CI: 1.6–2.6) for patients treated with BCS + RT and grade III DCIS versus 1.6% (95% CI: 0.94–1.7) patients with grade I/II DCIS treated with BCS + RT. For patients treated with BCS alone and grade III DCIS the cumulative incidence of iIBC was 5.3% (95% CI: 2.7–9.3), versus a cumulative incidence of 3.3% (95% CI: 2.2–4.7) in grade I/II DCIS patients at 5 years (Fig. 3).

**Table 1:** Characteristics of the study population by strategy

**Initial DCIS treatment**	BCS + RT	BCS alone	MST	Total
**Characteristics**	n (%)	n (%)	n (%)	n (%)
**Age at DCIS diagnosis (years)**				
< 40	120 (1.6)	30 (2.4)	325 (6.0)	475 (3.3)
40–49	767 (9.8)	169 (13.7)	921 (17.2)	1.857 (12.9)
50–59	3.074 (39.3)	487 (39.6)	1.966 (36.6)	5.527 (38.3)
60–69	2.767 (35.4)	308 (25.0)	1.379 (25.7)	4.554 (30.9)
70–79	1.051 (13.4)	167 (13.6)	657 (12.2)	1.875 (13.0)
> 80	40 (0.5)	70 (5.7)	121 (2.3)	231 (1.6)
Median (interquartile range)	59 (52–66)	57 (51–67)	56 (50–65)	58 (51–66)
**Period of DCIS diagnosis**				
2005–2009	2.557 (32.7)	427 (34.7)	2.158 (40.2)	5.124 (35.7)
2010–2015	5.262 (67.3)	804 (65.3)	3.211 (59.8)	9.277 (64.3)
**Screen-detected**				
Yes	3.699 (47.3)	482 (39.1)	1.693 (31.5)	5.874 (40.7)
No	941 (12.0)	193 (15.7)	1.132 (21.1)	2.266 (15.7)
Missing	3.179 (40.7)	556 (39.1)	2.544 (47.4)	6.279 (43.6)
**DCIS grade**				
I	1.070 (13.7)	626 (50.9)	496 (8.8)	2.165 (15.0)
II	2.814 (36.0)	274 (22.2)	1.558 (29.0)	4.646 (32.2)
III	3.675 (47.0)	190 (15.4)	3.173 (59.1)	7.038 (48.8)
Missing	260 (3.3)	141 (11.5)	169 (3.1)	570 (4.0)
**Follow-up interval (years)**				
0–5	1.098 (14.1)	250 (20.3)	698 (13.0)	2.046 (14.2)
5–10	4.366 (55.8)	616 (50.0)	2.717 (50.6)	7.699 (53.4)
> 10	2.355 (30.1)	365 (26.7)	1.954 (36.4)	4.674 (32.4)
Median (interquartile range)	8.0 (5.9–10.7)	7.6 (5.3–10.5)	8.6 (6.2–11.3)	8.2 (6.0-10.9)
**Subsequent iIBC**				
No	7.617 (97.4)	1.162 (93.4)	5.291 (98.5)	14.070 (97.6)
yes	202 (2.6)	69 (6.6)	78 (1.5)	349 (2.4)
**Total**	7.819	1.231	5.369	14.419

BCS, breast conserving surgery; RT, radiotherapy; MST, Mastectomy; n, number, iIBC ipsilateral invasive breast cancer

Analysis showed that hazard rates for the association of treatment with iIBC risk was non proportional over time. Therefore, the final model included a cross product of treatment type and time. In the multivariable analysis, patients treated by BCS alone had a 2.80 (95% CI: 1.91–4.10) higher risk of developing iIBC compared to BCS + RT, whereas patients treated with MST had a HR of 0.70 (95% CI:0.50–0.99) for developing iIBC compared to BCS + RT within the first 5 years after primary treatment. After 5 years, the risk of iIBC remained 1.73 (95% CI: 1.15–2.61) times higher for BCS alone compared to BCS + RT whereas, for the MST treated patients, the hazard-ratio further decreased (HR 0.30; 95% CI: 0.20–0.40), see Table 2. There was no significant interaction of DCIS grade with treatment type and DCIS grade was no confounding factor in the association of treatment type with the risk of iIBC.

**Table 2:** Multivariable Cox regression analysis for iIBC in women treated for DCIS

Follow-up time (years)	Treatment	iIBCs number	HR (95% CI)*	p-value
0–5	BCS + RT	89	ref	
	BCS alone	40	2.80 (1.91–4.10)	< 0.001
	MST	52	0.70 (0.50–0.99)	0.046
≥ 5	BCS + RT	113	ref	
	BCS alone	29	1.73 (1.15–2.61)	0.008
	MST	26	0.30 (0.20–0.40)	< 0.001
**Per age group**				
< 50 years				
0–5	BCS + RT	14	ref	
	BCS alone	10	3.20 (1.41–7.18)	0.005
	MST	32	1.40 (0.73–2.62)	0.308
≥ 5	BCS + RT	24	ref	
	BCS alone	6	1.12 (0.50–2.80)	0.799
	MST	8	0.20 (0.08–0.42)	< 0.001
≥ 50 years				
0–5	BCS + RT	75	ref	
	BCS alone	30	2.80 (1.82–4.30)	< 0.001
	MST	20	0.44 (0.30–0.73)	0.002
≥ 5	BCS + RT	89	ref	
	BCS alone	23	1.94 (1.22–3.10)	0.005
	MST	18	0.30 (0.20–0.50)	< 0.001

*With age as primary time-scale, and treatment as time-varying variable

iIBC, ipsilateral invasive breast cancer; HR, hazard ratio; CI, confidence interval; BCS, breast-conserving surgery; RT, radiotherapy; MST, mastectomy.

## Discussion

Here we show a low absolute risk for a subsequent iIBC at 10-year after a diagnosis and treatment of primary DCIS without invasive breast cancer. With a median time to iIBC of 4.8 years and median follow-up of 8.2 years from patients diagnosed with DCIS from 2005 to 2015, the cumulative incidences of subsequent iIBC are 3.1% after BCS + RT, 7.3% after BCS alone and 1.6% after MST. Although absolute risks of iIBC are low in patients treated for DCIS by either BCS and or BCS + RT, the risk remained higher for patients treated by BCS alone compared to patients treated with BCS + RT for at least 10 years after DCIS diagnosis. Compared to our previous study of van Seijen et al. [15], which included 10,045 primary DCIS patients diagnosed from 1989 to 2004 the current study reports lower absolute risks for iIBCs for the different treatment strategies for primary DCIS. Van Seijen et al. reported 10-year cumulative incidences of 5.2% after BCS + RT, 13.9% after BCS alone and 1.1% after MST with a median follow-up of 15.7 years after diagnosis. Comparing the 10 years cumulative incidences of our previous study to the current study, a reduced risk of approximately 50% for the different treatment strategies, with exception of the MST treated group, is demonstrated. In addition, trends of decreasing hazard ratios over time in the current study were also seen, similar to those reported by van Seijen et al. [15]. The current study more accurately reflects the daily practice in managing DCIS nowadays, since patients included in this study were diagnosed from 2005 to 2015. Current practice comprises a fully implemented Dutch breast cancer screening program and the addition of RT in standard care for DCIS in case of BCS [21]. Luijten et al. [22], demonstrated the patterns of treatment in DCIS patients over time since the introduction of breast cancer screening in the Dutch population. They showed that use of BCS increased from 47.7% in 1995–1996 to 72.7% in 2017–2018. Also, a sharp rise in the use of adjuvant radiotherapy in patients treated with BCS was observed, from 28.9% in 1995–1996 to almost 90% in 2011–2012, followed by a drop to 74.9% in 2017–2018. The addition of radiotherapy could be an explanation for the lower absolute risks for subsequent iIBC as 86.4% of the patients treated with BCS received adjuvant RT compared to just 49.6% of patients from our previous study [14]. The decline in risk of a subsequent breast event after a diagnosis of DCIS over time has been observed in two earlier studies as well [16, 17]. Halasz et al. reported on 246 consecutive patients who underwent BCS and RT for DCIS from 2001 to 2007 and attributed the risk decline to improved resection margins and better detection in modern era mammography [17]. Subhedar et al. retrospectively reviewed a prospectively collected cohort of 2.996 DCIS patients with a median follow-up of 6.3 years treated with BCS from the years 1978–2010 and observed similar declines in the risk of a subsequent breast event with later year of DCIS diagnosis. They concluded that the decline in subsequent breast events after DCIS could only partially be explained by the increased proportion of screen-detected patients, more clear margins, and the increased use of RT [16]. In our study no information was available regarding resection margins, and since in the Netherlands patients do not receive adjuvant endocrine therapy, this was not a possible factor influencing risk of iIBC. Our study did not take into account the non-invasive recurrences. Although they are clinically of less important, these lesions may have a severe impact on patient. Additionally, we investigated whether cumulative incidences in patients low-grade DCIS versus high-grade DCIS showed strong differences. However, these results showed only marginally and clinically non-significant differences (see Figs. 2 and 3).

This study has several strengths and limitations. A limitation of this study is the potential of confounding by indication, considering that women with less favorable characteristics more probably received more invasive treatment in terms of adjuvant radiotherapy which may have resulted in an underestimation of the difference in iIBC risk between BCS + RT and BCS alone. Furthermore, risk factors for developing iIBC such as primary lesion size and margin status could not be studied since information was not available. However, the magnitude of these risk factors associated with a subsequent iIBC after DCIS is still debated [23, 24].

An important strength of this study is that the included population is reflective of the current management of DCIS since adjuvant RT was incorporated as standard care for DCIS, ensuring a homogeneous study population. Also, over the years, more detailed data have been registered by NCR and PALGA, enabling more complete datasets. For this study both the NCR- and PALGA-data were scrutinized to identify primary DCIS patients, providing a true primary pure DCIS cohort. The nationwide NCR registers all primary DCIS patients in the Netherlands as of 2001 and includes both screen-detected and non-screen detected DCISs. Therefore, this dataset is unique with regard to its size and the robustness of the data due to the comprehensive registration of DCIS and IBC.

In conclusion, we report low absolute risks of iIBC after diagnosis of DCIS. These results are in line with more recently reported declining trends of a subsequent iIBC after DCIS. Possible explanations for this declining trend might be the more frequent use of adjuvant RT, an increased proportion of radical resection, and a higher proportion of screen-detected DCIS. The very low risk of an invasive recurrence observed in this study supports current efforts in active surveillance trials to determine whether it is safe to omit loco-regional treatment in patients with lower-grade (grade I/II) DCIS [25–27]. For high-grade DCIS the results of this study warrant a further exploration of omission of radiotherapy in selected patients.

## Data Availability

The datasets generated and/or analyzed during the current study are not publicly available because consent was not obtained from study participants to make these data publicly available, but de-identified data are available from the corresponding author on reasonable request.
